# Treatment of anorexia nervosa with long-term risperidone in an outpatient setting: case study

**DOI:** 10.1186/2193-1801-3-706

**Published:** 2014-12-02

**Authors:** Elsa J Kracke, Aneesh K Tosh

**Affiliations:** University of Missouri School of Medicine, One Hospital Drive, MA204, DC018.00, Columbia, MO 65212 USA; University of Missouri Department of Child Health, 400 N. Keene Street, Columbia, MO 65201 USA

**Keywords:** Anorexia nervosa, Risperidone, Secondary amenorrhea, Eating disorders, Outpatient treatment, Case report

## Abstract

**Introduction:**

There are currently few studies focusing on the efficacy of long-term atypical antipsychotics to treat anorexia nervosa in the pediatric population.

**Case description:**

This case report follows the treatment of a 17 year-old female with anorexia nervosa over her four-year undergraduate career. After two years of multidisciplinary treatment, low-dose risperidone was initiated due to persistence of her disease. She expressed decreased rigidity around meal times, her weight improved and she had resumption of menses. She was compliant with treatment through graduation and maintained her weight gain.

**Discussion & evaluation:**

Atypical antipsychotics are a treatment option in the management of anorexia nervosa. Risperidone has not been studied as frequently as olanzapine for eating disorders. Risperidone was chosen for its more favorable side effect profile and decreased cost to the patient. Previous studies on anorexia nervosa treatment have occurred during inpatient treatment and have limited follow-up due to patients’ refusal to initiate or maintain medication compliance. This case presents 17 months of outpatient data. The efficacy of risperidone therapy was evaluated with frequent weight checks, subjective decrease in rigidity, serial complete metabolic panels, and restoration of menses.

**Conclusions:**

In this case report, an adolescent female treated with low-dose risperidone had decreased rigid thinking, weight gain and resolution of secondary amenorrhea without medication side effects. Therefore, the atypical antipsychotic risperidone may be an effective long-term outpatient treatment option for patients with anorexia nervosa.

## Introduction

Anorexia nervosa (AN) restricting subtype is defined as an individual that loses weight through dieting, fasting and/or excessive exercise over at least a three-month consecutive period. Characteristics of AN, such as the intense fear of gaining weight, disturbance in the perception of body shape and denial of the seriousness of extremely low body weight, have been compared to the signs of rigidity in psychosis and inflexibility of delusional disorders. Comorbid depression is also often seen in patients with AN. Therefore, atypical antipsychotics that act on both 5-HT and dopamine receptors have recently been studied to monitor improvements in behavior and weight gain (Hagman et al. 2011). In addition, the minimal sedating effects of atypical antipsychotics may benefit patients that have concomitant anxiety, and could reduce or replace the use of SSRIs in the treatment of AN. Antidepressants have not been successful for weight gain in AN, yet are used to treat associated co-morbid conditions such as depression and anxiety. Current literature focuses primarily on short-term olanzapine use in patients with AN while they are admitted for inpatient therapy. This case report is a unique presentation of a female with AN that accomplished weight restoration with resumption of menses after long-term treatment with risperidone completely in an outpatient setting.

## Case description

This case report follows the four-year treatment of patient that presented at the beginning of her freshman year of college to a university health clinic with an established diagnosis of anorexia nervosa. She was initially diagnosed in January 2008, at 17 years of age. Her premorbid weight was 50 kg. Her menses previously had cycle length irregularity, however April 2008 marked her last menstrual period. When she presented for her first visit to the health center on August 18, 2008, she weighed 41.36 kg at a height of 172.72 cm (BMI 13.8 kg/m2) and had developed secondary amenorrhea. Her eating disorder initially started by restricting fats, and progressed to eliminating all fats and then overall calories. These characteristics are consistent with AN restrictive subtype. The patient did not report purging, laxative abuse or excessive exercising to compensate for calorie intake. Although she played competitive tennis in high school, she reduced her activity level during college considerably.

She noted anxiety and fatigue in review of systems, and denied signs of depression. No significant past medical history, surgical history or family history, including history of eating disorders or other psychiatric conditions, was mentioned during her treatment. Her medication list when she presented consisted of escitalopram for anxiety, doxycycline for acne, a generic multivitamin and a fish oil supplement. Significant findings on initial physical exam were a thin-appearing female with lanugo. Vital signs revealed sinus bradycardia with a heart rate of 56 bpm, blood pressure 102/60, temperature 97.3 F and BMI of 13.8 kg/m2.

## Treatment course

The patient presented in August 2008 at 41.36 kg already taking escitalopram 20 mg. In the 24 days following her presentation, she proceeded to lose weight until she reached 39.32 kg (BMI 13.2 kg/m2). At this time, the multidisciplinary medical team made the decision for her to enter residential treatment at an outside eating disorder treatment facility. After 3 months of structured inpatient treatment, her weight increased to 47.73 kg, and she was able to enroll in classes for the spring semester. Over the next 10 months she had no significant improvement in weight or mealtime anxiety. Escitalopram was discontinued and replaced with sertraline at 50 mg. The dose of sertraline gradually increased from 50 mg to 75 mg to 100 mg and then to a maximum of 150 mg over a one-year period to treat her anxiety. Throughout this time, the patient still had subjective feelings of anxiety and rigidity at meal times and therefor did not have significant improvement in weight gain. On November 29, 2010, at a weight of 46.64 kg, she agreed to begin a low-dose atypical antipsychotic, risperidone 0.25 mg. Approximately 3 months later, she had gained only1.36 kg and reported no medication side effects, so the dose of risperidone was increased to 0.5 mg. After seven months of compliance at this dose, she gained another 6 kg and was able to decrease her sertraline dose to 100 mg.

Lab work during the two years from initial presentation to the addition of risperidone (2008–2010) showed a complete metabolic panel within normal limits and a normal vitamin D level at 37 ng/mL. Electrocardiogram showed sinus bradycardia without QTc prolongation. Complete metabolic panel, fasting lipid panel and prolactin lab values two years after starting risperidone continued to be within normal limits (Table 1), and electrocardiogram showed sinus rhythm without QTc prolongation.

**Table 1:** **Lab values 1/27/2012**

**Fasting lipid panel**	
Total cholesterol	185 mg/dL
Triglycerides	100 mg/dL
HDL	55 mg/dL
LDL	110 mg/dL
**Complete metabolic panel**	
Glucose	93 mg/dL
AST	22 U/L
ALT	21 U/L
**Prolactin**	8.2 ng/mL

**Figure 1:**
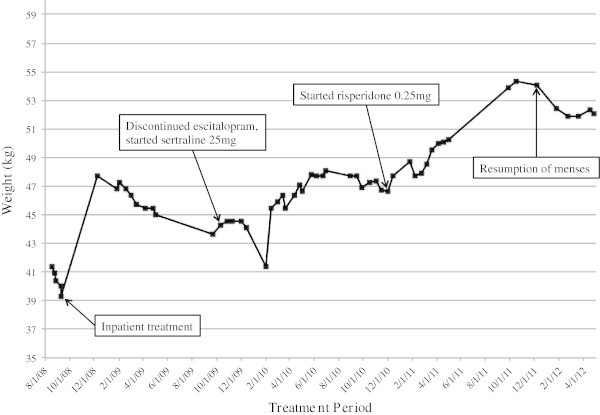
**Weight gain throughout four-year treatment period.**

In December 2011, she had resumption of menses at a weight of 54.09 kg (BMI 18.1 kg/m2). Her weight remained stable, and the last weight recorded in April 2012 was 52.09 kg (BMI 17.5 kg/m2) (Figure 1, Table 2). Her care was transferred after graduation from university, and she continues to remain in recovery.

**Table 2:** **Significant events during treatment**

Date	Weight (kg)	Event
8/18/08	41.36	First clinic visit
9/11/08	39.32	Inpatient treatment at an outside treatment facility (88 days)
12/8/08	47.73	Escitalopram 20 mg
10/12/09	44.32	Discontinued escitalopram, started sertraline 25 mg
10/26/09	44.55	Increased sertraline 50 mg
11/30/09	44.55	Increased sertraline 75 mg
9/13/10	47.73	Increased sertraline 100 mg
11/1/10	47.36	Increased sertraline 150 mg
11/29/10	46.64	Started risperidone 0.25 mg, continued sertraline 150 mg
2/21/11	47.91	Risperidone increased to 0.5 mg, continued sertraline 150 mg
9/26/11	53.91	Sertraline decreased to 100 mg, continued risperidone 0.5 mg
12/5/11	54.09	Restoration of menses
4/27/12	52.09	Last clinic visit

## Discussion and evaluation

This case presents a patient with anorexia nervosa that was treated over the course of her four-year undergraduate career primarily in an outpatient setting. After two years of multidisciplinary treatment with a physician, a dietitian, a therapist and taking an SSRI, she decided to start risperidone. Although many studies have focused on olanzapine for weight gain, risperidone was chosen for its more favorable side effect profile and the significant decreased cost to the patient. The patient’s lab work remained within normal limits after initiating treatment, suggesting a low dose atypical antipsychotic could provide the benefit of gradual weight gain without metabolic consequences. Reduced cost to the patient may not only increase compliance and initial willingness to try the medication, but also justifies the use of risperidone instead of olanzapine as a long-term therapy to maintain weight gain and reach a recovery phase.

Near the end of her treatment, after resumption of menses, the patient was queried as to why she felt the risperidone was helpful in her recovery. The patient felt that she had a significant improvement in rigidity during meal times after starting the risperidone. Despite having similar motivation to improve her nutrition in the past, the rigidity was a barrier that she was not able to overcome until the reperidone was started. Also, it is well known that weight gain is a side effect of risperidone therapy. While her metabolic labs were normal and stable during her treatment, the possibility that weight gain as a side effect of risperidone contributed to her resumption of menses is another conceivable factor in her recovery. Therefore, a combination of factors related to the risperidone likely played a role in the patient’s recovery.

This case is unique because the patient had good insight and was motivated to gain weight, resulting in strict compliance with her medical treatment. The patient was highly motivated to attend college and had strong support from her parents and friends relative to other patients treated in the eating disorders clinic. However, these impressions are subjective. Therefore, one limitation to this report is that personal events throughout this patient’s undergraduate experience that may have influenced her improved health are not quantifiable. Previous studies focused on treating AN tend to be limited due to the ego-syntonic quality of AN and the refusal to start or maintain treatment for fear of weight gain as a medication side effect (Balestrieri et al. 2013). For this reason, replicating the compliance and success demonstrated in this case report might be difficult in a patient unmotivated to gain weight. For patients noncompliant with oral medication, risperidone long-acting injection may be another beneficial alternative (Umehara et al. 2014). Not only was this patient able to gain weight with risperidone, she experienced decreased rigidity around meal times and was able to decrease her SSRI dose for anxiety. Previously published studies, with one exception (Hagman et al. 2011), have not treated AN with concomitant SSRI and atypical antipsychotics, and this is the only report to our knowledge of decreasing the SSRI dose during the treatment period. Resumption of menses after one year on risperidone was another measure of improvement that has been mentioned only one time previously in the literature (Newman-Toker 2000). The patient had experienced secondary amenorrhea for 3 years and 8 months before the return of her menses. This case also represents the longest published treatment with an atypical antipsychotic, 17 months, in addition to being completely in an outpatient setting. Therefore, risperidone may become a feasible and effective outpatient option for patients with AN. She experienced no side effects from her medications, and when asked which therapy helped her the most, she contributed her successful weight gain to risperidone.

## Conclusions

This case report demonstrates that the atypical antipsychotic risperidone could be a safe and effective option for adolescent patients with anorexia nervosa motivated to gain weight. Ideally, the next steps would be to recruit more patients with anorexia nervosa to participate in a randomized controlled trial to determine the efficacy of multidisciplinary therapy with risperidone or placebo in the outpatient setting. As previously mentioned, recruiting patients with anorexia nervosa interested in participating in studies on therapy options is challenging.

## Authors’ information

AKT is a board-certified Adolescent Medicine physician at the University of Missouri with an interest in Eating Disorders. EJK is a fourth-year medical student pursuing a pediatrics residency position.

## Abbreviations

AN: Anorexia nervosa
SSRIs: Selective serotonin reuptake inhibitors
BMI: Body mass index.
